# Low-level expression of SAMHD1 in acute myeloid leukemia (AML) blasts correlates with improved outcome upon consolidation chemotherapy with high-dose cytarabine-based regimens

**DOI:** 10.1038/s41408-018-0134-z

**Published:** 2018-10-19

**Authors:** George Z. Rassidakis, Nikolas Herold, Ida Hed Myrberg, Nikolaos Tsesmetzis, Sean G. Rudd, Jan-Inge Henter, Torsten Schaller, Siok-Bian Ng, Wee Joo Chng, Benedict Yan, Chin Hin Ng, Farhad Ravandi, Michael Andreeff, Hagop M. Kantarjian, L. Jeffrey Medeiros, Ioanna Xagoraris, Joseph D. Khoury

**Affiliations:** 1 Department of Oncology-Pathology, Karolinska Institutet and Karolinska University Hospital, Stockholm, Sweden; 2 Childhood Cancer Research Unit, Department of Women’s and Children’s Health, Karolinska Institutet and Karolinska University Hospital, Stockholm, Sweden; 3 Science for Life Laboratory, Department of Oncology-Pathology, Karolinska Institutet, Stockholm, Sweden; 4 Department of Infectious Diseases, Virology, University Hospital Heidelberg, Heidelberg, Germany; 5 National University Cancer Institute of Singapore, Singapore, Singapore; 6 Department of Pathology, National University Hospital and Yong Loo Lin School of Medicine, National University of Singapore, Singapore, Singapore; 7 Department of Leukemia, The University of Texas MD Anderson Cancer Center, Houston, TX, USA; 8 Department of Hematopathology, The University of Texas MD Anderson Cancer Center, Houston, TX USA

## Abstract

Sterile alpha motif and histidine/aspartic acid domain containing protein 1 (SAMHD1) limits the efficacy of cytarabine (ara-C) used in AML by hydrolyzing its active metabolite ara-CTP and thus represents a promising therapeutic target. SAMHD1 has also been implicated in DNA damage repair that may impact DNA damage-inducing therapies such as anthracyclines, during induction therapy. To determine whether SAMHD1 limits ara-C efficacy during induction or consolidation therapy, SAMHD1 protein levels were assessed in two patient cohorts of *de novo* AML from The University of Texas MD Anderson Cancer Center (USA) and the National University Hospital (Singapore), respectively, using immunohistochemistry and tissue microarrays. SAMHD1 was expressed at a variable level by AML blasts but not in a broad range of normal hematopoietic cells in reactive bone marrows. A sizeable patient subset with low SAMHD1 expression (<25% of positive blasts) was identified, which was significantly associated with longer event-free (EFS) and overall (OS) survival in patients receiving high-dose cytarabine (HDAC) during consolidation. Therefore, evaluation of SAMHD1 expression level in AML blasts at diagnosis, may stratify patient groups for future clinical trials combining HDAC with novel SAMHD1 inhibitors as consolidation therapy.

## Introduction

Acute myeloid leukemia (AML) is a heterogeneous group of neoplasms derived from myeloid progenitor cells. Overall survival (OS) after five years in AML patients is age-dependent and ranges from ~70% in children^1^ to less than 20% in elderly adults^2^. The most important drugs in the treatment of AML patients are anthracyclines that contribute heavily to the success of remission induction therapy^3^ and cytarabine (ara-C). Ara-C is particularly effective in high-dose remission consolidation courses^4^. The inter-patient variability of response to high-dose ara-C (HDAC) regimens correlates with the propensity of AML blasts to accumulate ara-CTP intracellularly^5^, the main determinant of ara-C efficacy^6^.

We and others recently identified SAMHD1 as a major negative factor limiting ara-CTP accumulation and retention via a hitherto unknown ara-CTPase activity^7–12^. SAMHD1 decreases intracellular ara-CTP concentrations, limiting its lethal mis-incorporation into DNA and thus promoting cell survival^7,13.^ Ara-C treatment was more effective in AML xenotransplant mouse models lacking functional SAMHD1 as compared to SAMHD1-proficient counterparts^7,8,11^. Furthermore, depletion of SAMHD1 in primary AML blasts using the lentiviral protein X (Vpx), which targets SAMHD1 for degradation, increased ara-C sensitivity^7^.

In our previous report we were able to demonstrate that ara-C-treated patients with higher *SAMHD1* mRNA expression at diagnosis had reduced OS and event-free survival (EFS) as compared to patients with lower *SAMHD1* expression, in both the adult *de novo* The Cancer Genome Atlas (TCGA) and the pediatric Therapeutically Applicable Research to Generate Effective Treatments (TARGET) AML cohorts^7^. However, complete responders versus non-responders showed no significant difference in *SAMHD1* mRNA expression in either cohort^7^. By contrast, high SAMHD1 expression tended to result in better complete response rates^7^, which might be explained by the role of SAMHD1 as a tumor suppressor^9^. SAMHD1 also has been implicated in DNA repair and thus modulates the efficacy of DNA damage-inducing agents^14,15^. Hence, possible advantages of low SAMHD1 levels in patients treated with low-dose ara-C might be mitigated by its combination with anthracyclines, which would rather benefit from higher SAMHD1 expression^8,9,11^. In addition, we could demonstrate that the effect of SAMHD1 on AML survival varies with time after diagnosis^9^. Accordingly, for clinical trials aimed at adjusting ara-C doses according to SAMHD1 expression or to combine ara-C with methods to target SAMHD1, it is of utmost importance to understand when to expect the most benefit from these interventions. Dose adjustments and addition of potential strategies to inhibit SAMHD1 at the wrong time might jeopardize possible therapeutic improvements or lead to worse outcomes due to excess toxicity or inhibition of SAMHD1 tumor suppressor functions.

Toward this end, we analyzed two independent and clinically different cohorts of *de novo* AML to assess whether SAMHD1 protein expression in blasts at the time of diagnosis correlates with clinical endpoints, including complete remission (CR), EFS and OS, as well as the type of ara-C treatment (low-dose vs. high-dose). The data presented here suggest that SAMHD1 expression in AML blasts correlates with clinical outcome after HDAC consolidation therapy.

## Patients and methods

### Patient groups

Eligible patients had tissue specimens available for immunohistochemical determination of SAMHD1 expression. The diagnosis and subclassification of AML were established according to criteria defined in the World Health Organization classification (2008). A total of 222 *de novo* AML patients with available diagnostic bone marrow specimens were included, and immunohistochemistry was evaluable in 189 patients. Of these, 98 patients were diagnosed and treated at The University of Texas MD Anderson Cancer Center (MDACC) between 1 June 2007 and 31 December 2015 (87 of which had evaluable IHC results), and 124 patients were diagnosed and treated at the National University Hospital of Singapore (NUH) between 1 February 2001 and 31 December 2011 (102 of which had evaluable IHC results). Research use of these samples was in accord with the Declaration of Helsinki and was approved by the Institutional Review Boards of MDACC (USA) and Domain Specific Review Board, National Healthcare Group (Singapore). The basic demographic and clinical data, including therapy, for both cohorts are shown in Table 1. The cohorts from MDACC and NUH differed substantially with regards to the extent of SAMHD1 expression (percentage of positive blasts), cytogenetic risk group, treatment, response to therapy as well as *NPM1* mutation status (Table 1). These two completely different AML cohorts were included in the study on purpose in order to further validate the clinical significance of SAMHD1 expression in AML in various biological backgrounds and different clinical settings. Patients were allocated to favorable-, intermediate- and high-risk cytogenetic risk groups per currently used National Cancer Center Network (NCCN) guidelines (version 3, 2017).

**Table 1:** Characteristics of included patients with valid SAMHD1 data

	Total	MDACC	Singapore	*P*
Number of included patients	189	87	102	
Age (years), median (range)	52 (18–80)	56 (18–79)	48 (19–80)	0.054^a^
SAMHD1 percent positive blasts, median (range)	30 (1–100)	40 (3–100)	25 (1–95)	0.021^a^
Male sex, *n* (%)	103 (54.5)	50 (57.5)	53 (52.0)	0.47^d^
Cytogenetic risk group				<0.0001^b^
Favorable, *n* (%)	12 (6.3)	0 (0.0)	12 (11.8)	
Intermediate, *n* (%)	157 (83.1)	87 (100.0)	70 (68.6)	
Poor, *n* (%)	19 (10.1)	0 (0.0)	19 (18.6)	
N/A, *n* (%)	1 (0.5)	0 (0.0)	1 (1.0)	
Response				<0.0001^b^
Complete response, *n* (%)	106 (56.1)	74 (85.1)	32 (31.4)	
No complete response, *n* (%)	68 (36.0)	11 (12.6)	57 (55.9)	
N/A, *n* (%)	15 (7.9)	2 (2.3)	13 (12.7)	
FLT3-ITD mutation				0.56^b^
Positive, *n* (%)	45 (23.8)	32 (36.8)	13 (12.7)	
Negative, *n* (%)	84 (44.4)	54 (62.1)	30 (29.4)	
N/A, *n* (%)	60 (31.7)	1 (1.1)	59 (57.8)	
FLT3-TKD mutation				0.17^b^
Positive, *n* (%)	11 (5.8)	10 (11.5)	1 (1.0)	
Negative, *n* (%)	115 (60.8)	76 (87.4)	39 (38.2)	
N/A, *n* (%)	63 (33.3)	1 (1.1)	62 (60.8)	
NPM1 mutation				0.0075^b^
Positive, *n* (%)	44 (23.3)	38 (43.7)	6 (5.9)	
Negative, *n* (%)	60 (31.7)	37 (42.5)	23 (22.5)	
N/A, *n* (%)	85 (45.0)	12 (13.8)	73 (71.6)	
IDH mutation				
Positive, *n* (%)	11 (5.8)	0 (0.0)	11 (10.8)	
Negative, *n* (%)	37 (19.6)	0 (0.0)	37 (36.3)	
N/A, *n* (%)	141 (74.6)	87 (100.0)	54 (52.9)	
DNMT3A mutation				
Positive, *n* (%)	10 (5.3)	0 (0.0)	10 (9.8)	
Negative, *n* (%)	39 (20.6)	0 (0.0)	39 (38.2)	
N/A, *n* (%)	140 (74.1)	87 (100.0)	53 (52.0)	
Induction treatment				<0.0001^c^
High-dose AraC	89 (47.1)	87 (100.0)	2 (2.0)	
Low-dose AraC	55 (29.1)	0 (0.0)	55 (53.9)	
Other/N/A, *n* (%)	45 (23.8)	0 (0.0)	45 (44.1)	
Consolidation treatment				<0.0001^d^
High-dose AraC	119 (63.0)	87 (100.0)	32 (31.4)	
Other/N/A, *n* (%)	70 (37.0)	0 (0.0)	70 (68.6)	
Clofarabine				
Yes, *n* (%)	23 (12.2)	23 (26.4)	0 (0.0)	
No, *n* (%)	64 (33.9)	64 (73.6)	0 (0.0)	
N/A, *n* (%)	102 (54.0)	0 (0.0)	102 (100.0)	
Fludarabine				
Yes, *n* (%)	25 (13.2)	25 (28.7)	0 (0.0)	
No, *n* (%)	62 (32.8)	62 (71.3)	0 (0.0)	
N/A, *n* (%)	102 (54.0)	0 (0.0)	102 (100.0)	
Cladribine				
Yes, *n* (%)	2 (1.1)	2 (2.3)	0 (0.0)	
No, *n* (%)	85 (45.0)	85 (97.7)	0 (0.0)	
N/A, *n* (%)	102 (54.0)	0 (0.0)	102 (100.0)	
Sorafenib				
Yes, *n* (%)	6 (3.2)	6 (6.9)	0 (0.0)	
No, *n* (%)	81 (42.9)	81 (93.1)	0 (0.0)	
N/A, *n* (%)	102 (54.0)	0 (0.0)	102 (100.0)	
Gemtuzumab-ozogamicin				
Yes, *n* (%)	6 (3.2)	6 (6.9)	0 (0.0)	
No, *n* (%)	81 (42.9)	81 (93.1)	0 (0.0)	
N/A, *n* (%)	102 (54.0)	0 (0.0)	102 (100.0)	
Vorinostat (SAHA)				
Yes, *n* (%)	12 (6.3)	12 (13.8)	0 (0.0)	
No, *n* (%)	75 (39.7)	75 (86.2)	0 (0.0)	
N/A, *n* (%)	102 (54.0)	0 (0.0)	102 (100.0)	

^a^Mann–Whitney *U*-test

^b^Fisher’s exact test (excluding N/A category)

^c^Chi-square test

^d^Fisher’s exact test

### Therapy

Treatment was as follows: 144 patients received ara-C-based induction therapy, four received non-ara-C containing induction therapy, and 41 lacked clinical data on the induction regimen used. Among the 189 patients in this study group with evaluable SAMHD1 data, 66 underwent allogeneic stem cell transplantation (allo-SCT). Signed informed consents (MDACC) or waiver of consent (NUH) were obtained before all procedures and before the administration of all investigational therapy according to local practice guidelines.

Unless the clinical condition precluded intensive chemotherapy, patients were treated with ara-C and anthracycline-based therapies. Low-dose ara-C treatment was 100–200 mg/m^2^. Patients treated at MDACC were all enrolled in clinical trials NCT00542971 (*n* = 5), NCT00656617 (*n* = 15), NCT00422591 (*n* = 1), NCT01025154 (*n* = 4), NCT01019317 (*n* = 25), NCT01289457 (*n* = 45), and NCT02115295 (*n* = 3) that—in addition to anthracyclines and ara-C—evaluated the addition of other antimetabolites, such as clofarabine, fludarabine, and cladribine, as well as tyrosine kinase inhibitors (sorafenib), histone deacetylase inhibitors (vorinostat/SAHA), and anti-CD33 antibodies (gemtuzumab-ozogamicin). CR was defined as absence of disease for at least 1 month as determined by physical examination, lack of blasts in the peripheral blood and blast counts below 5% in bone marrow aspirate smears. Partial remission (PR) was defined as at least a 50% decrease in the percentage of blasts in bone marrow aspirate smears to 5–25% of cellularity. Primary treatment failure was defined as failure to achieve CR during initial therapy. Relapse was defined as progression occurring at least 1 month after CR or PR.

### Construction of tissue microarrays (TMA) and immunohistochemical methods

All tissue samples were diagnostic bone marrow biopsy specimens obtained prior to induction therapy. The TMA were constructed using duplicate tumor cores selected from representative areas rich in blasts in each specimen. In addition, full tissue sections from five reactive bone marrow specimens as well as sections from formalin-fixed, paraffin-embedded cell blocks prepared from AML cell lines (Supplementary Methods) were included as controls. All immunohistochemical analyses were performed in the same research laboratory (Karolinska Institutet) using identical protocols for all TMAs, control bone marrow specimens and cell blocks after antigen retrieval as described previously^16^. A polyclonal rabbit anti-SAMHD1 antibody (cat. no. A303-691A, Bethyl Laboratories, San Antonio, TX, USA) and an automated detection system (Ventana Medical Systems, Roche, Basel, Switzerland) were utilized. The specificity of the polyclonal antibody was previously tested by Western blot analysis in AML cell lines and by immunohistochemistry using paraffin-embedded cell blocks as shown in Suppl. Figure 1. In addition to immunohistochemical detection of SAMHD1, double immunostainings were performed on reactive bone marrow specimens using four combinations of antibodies including SAMHD1/MPO, SAMHD1/GPA, SAMHD1/CD61, and SAMHD1/CD34 (Cat. no. 760-2659 for MPO, 760-4257 for GPA, 760-4249 for CD61, 790-2927 for CD34, all from Ventana, Roche Diagnostics Scandinavia, Stockholm, Sweden) in order to assess SAMHD1 expression in normal hematopoietic cells. In selected CD34+ AML cases, SAMHD1/CD34 double immunostaining was performed as well. SAMHD1 expression was restricted to the nuclei of blasts and positivity was defined as any level of unequivocal staining. Evaluation of the immunostained slides was blinded to any clinical data. At least 500 blasts were counted in each case, and the level of SAMHD1 expression was defined as the percentage of positive blasts. Based on visual inspection of the frequency distribution of the proportions of SAMHD1-positive AML blasts (histogram) in both the MDACC and NUH databases, two cutoffs were arbitrarily chosen to define a low (<25%) versus high (>75%) level of SAMHD1 expression as described below. Survival analysis also included the intermediate group with a percentage of SAMHD1-positive blasts between 25 and 75%.

### Statistical analysis

EFS was measured (number of days) from the beginning of treatment to death, relapse, or last follow-up. OS was measured (number of days) from the beginning of treatment to the time of last follow-up or death from any cause. As the present study investigated effects of SAMHD1 on induction and consolidation treatment, patients receiving allogeneic stem cell transplantation (allo-SCT) were censored on the date of transplant. Survival was visualized using Kaplan–Meier curves, and compared between groups of patients using the log-rank test. Cox proportional hazards models were fitted in order to evaluate the percentage of SAMHD1 positive blasts as a continuous predictor, and to adjust for other covariates. Interaction terms between SAMHD1 and clofarabine, and fludarabine, were tested but found non-significant, and were thus not included in the final models. Associations between SAMHD1 percent positive blasts and presenting clinical and laboratory features were evaluated using Spearman’s rank-correlation coefficient and the Mann–Whitney *U*-test, for continuous and dichotomous variables, respectively. Kruskal–Wallis test was used to test differences in percentage of SAMHD1 positive blasts between the three risk groups in the Singapore cohort. Presenting clinical and laboratory features were compared between the two patient cohorts from MDACC and NUH, respectively, using the Mann–Whitney *U*-test for continuous variables, and the Chi-square test or Fisher’s exact test for categorical variables, as appropriate. All statistical analyses were performed using R version 3.3.2 (The R Foundation for Statistical Computing, Vienna, Austria).

## Results

### Expression of SAMHD1 in reactive bone marrows and AML blasts

**Fig. 1:**
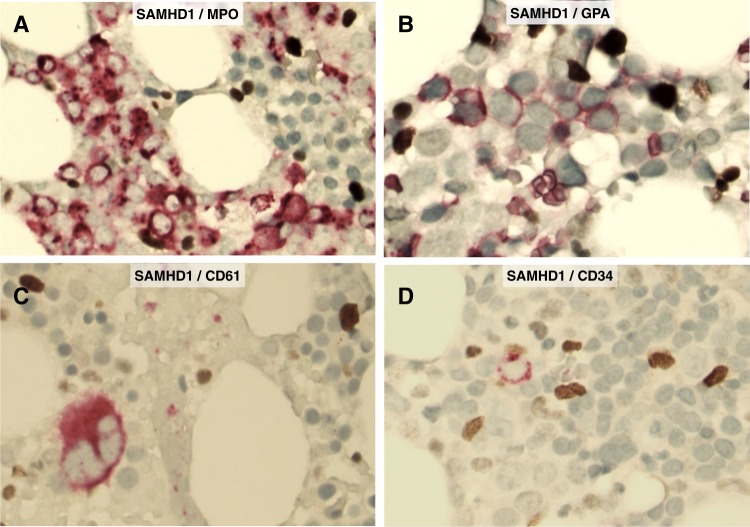
Expression of SAMHD1 protein in normal bone marrow. Double immunohistochemical staining shows that SAMHD1 protein is not detected in hematopoietic stem cells and progenitors (CD34+), cells of the myeloid/erythroid lineage (MPO+ or GPA+), and megakaryocytes (CD61+). Small reactive T-lymphocytes and macrophages are strongly positive for SAMHD1, thus serving as internal positive controls in each experimental setting (original magnification ×400; SAMHD1 in dark brown; MPO, GPA, CD61 and CD34 in red)

**Fig. 2:**
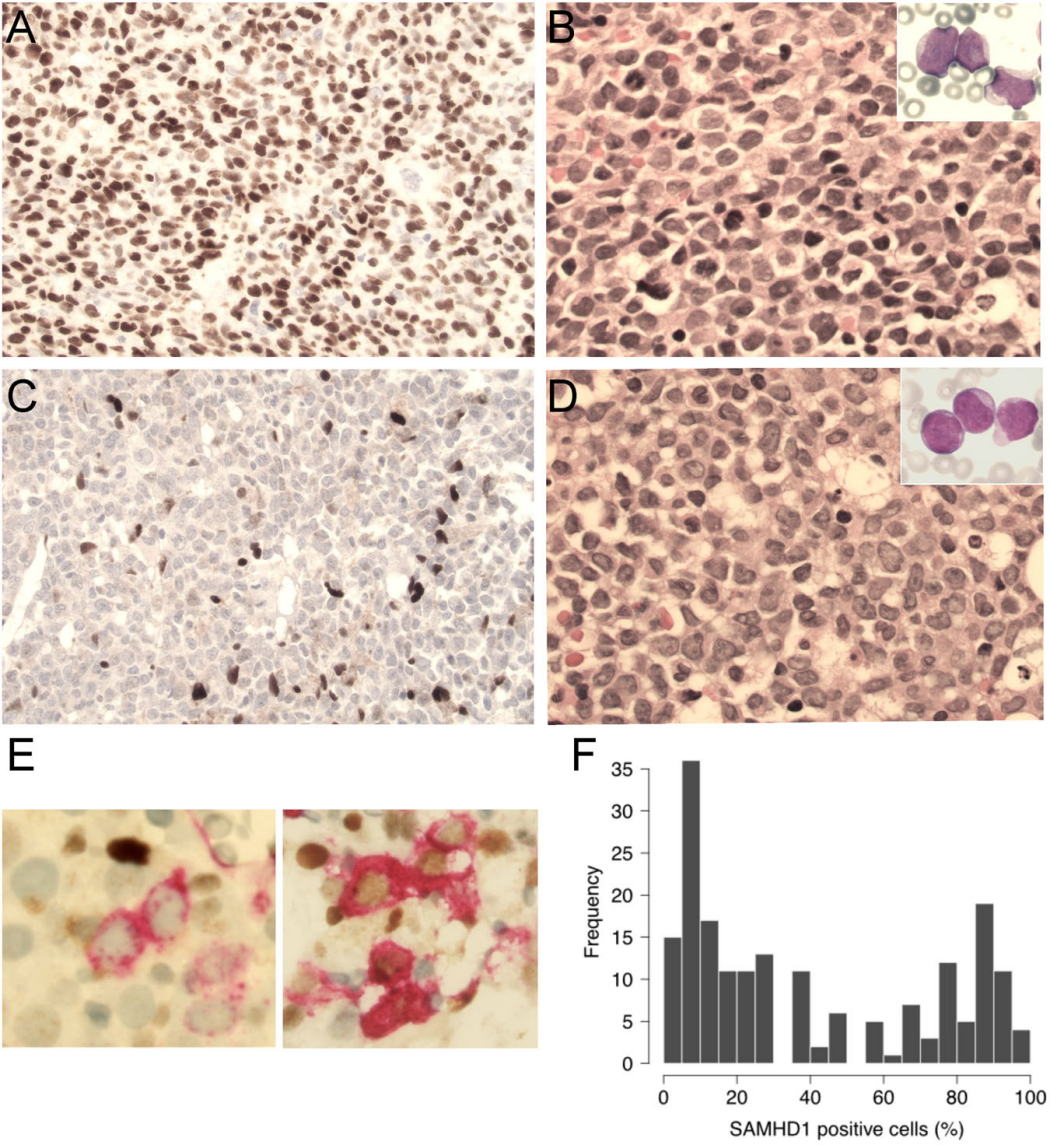
Expression of SAMHD1 protein in AML blasts. **a**, **b.** SAMHD1 is predominantly expressed in the nucleus of AML blasts. A representative AML case with high SAMHD1 expression (>75%) (**a**). Morphology of blasts is shown by routine hematoxylin-eosin (H&E) stain of bone marrow section (**b**) and by May-Grünwald Giemsa (MGG) stain of bone marrow smear (**b**, inset) (original magnification ×400 for **a**, **b** (and ×600 for **b,** inset). **c**, **d.** A representative case of AML with low SAMHD1 expression (<25%) (**c**). Morphology of blasts is shown by H&E stain of bone marrow section (**d**) and by MGG stain of bone marrow smear (**d**, inset) (original magnification ×400 for **c**, **d** and ×600 for **d**, inset). **e** Examples of double immunostainings (SAMHD1/CD34) in two CD34+ AML cases, one with SAMHD1-/CD34+ blasts (left) and another with SAMHD1+/CD34+ blasts (right). (original magnification ×600; SAMHD1 in dark brown; CD34 in red). **f** Histogram showing the distribution of SAMHD1 expression level (% of positive blasts) in TMAs of AML patients

In reactive bone marrows (free of malignancy), double immunohistochemistry demonstrated that normal hematopoietic stem cells and progenitors (CD34+), cells belonging to the myeloid/erythroid lineage (MPO+ or GPA+) as well as megakaryocytes (CD61+) were all negative for SAMHD1 expression (Fig. 1). In AML, SAMHD1 was detected predominantly in the nuclei of the blasts (Fig. 2a, c). In the entire study group, the median and mean percentage of the SAMHD1-positive blasts were 30% and 42%, respectively, and ranged from 1 to 100%. The percentage of SAMHD1-positive blasts differed significantly between the two cohorts (median 40% in MDACC vs. 25% in NUH, *P* = 0.021), but not between risk groups of the NUH cohort (*P* = 0.5266). The distribution of SAMHD1-positive blasts at diagnosis is shown as a histogram in Fig. 2f. Based on visual inspection of the distribution of these data, a 25% cutoff was chosen arbitrarily to define low SAMHD1 levels and a 75% cutoff to define high SAMHD1 levels. The associations between the levels of SAMHD1 (% positive AML blasts) and presenting clinical and laboratory features of the entire patient group are shown in Table 2. As a continuous variable, the percentage of SAMHD1-positive blasts was significantly associated with serum LDH levels and the white blood count, but not with blast count, CD34 expression in the blast population as assessed by flow cytometry and mutational status of *FLT3*, *NPM1*, *IDH*, or *DNMT3A* (Table 2). Importantly, no correlation between the percentage of SAMHD1-positive blasts at diagnosis and the subsequently administered ara-C dose was observed (Table 2).

**Table 2:** Association of SAMHD1 protein levels (% positive AML blasts) with patient characteristics at diagnosis

	Spearman correlation coefficient	*P*
Age (years)	0.081	0.27
PB blast count	0.10	0.34
BM blast count	−0.0061	0.94
WBC	0.230	0.034
Platelets	−0.15	0.17
Hemoglobin	0.091	0.40
LDH	0.36	0.00056
CD34	−0.10	0.37
FLT3-ITD mutation status		0.91^a^
FLT3-TKD mutation status		0.20^a^
NPM1 mutation status		0.36^a^
IDH mutation status		0.10^a^
DNMT3A mutation status		0.27^a^
Gender		0.63^a^
AraC dose^b^		0.40^a^

^a^Mann–Whitney *U*-test

^b^1.0 g × 5 days vs 1.5 g × 4 days

### Association of SAMHD1 expression levels with achievement of CR

**Fig. 3:**
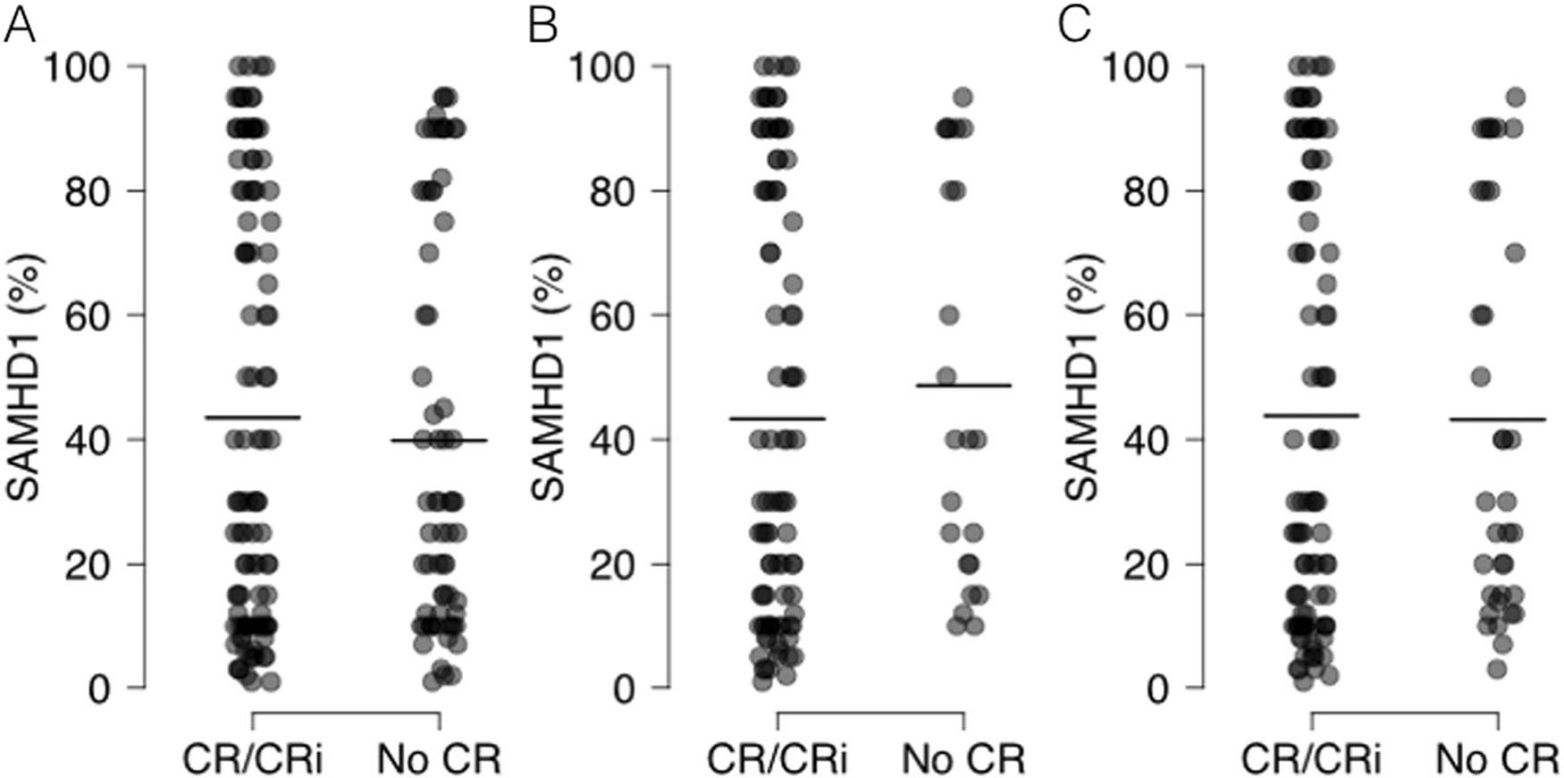
Expression of SAMHD1 in AML blasts in the groups of patients that achieved CR/CRi or not. The black lines indicate the mean percentage of SAMHD1 positive AML blasts. **a** All patients; **b** patients that received HDAC consolidation therapy; **c** patients that received ara-C-containing induction therapy

Following induction chemotherapy, 101 (53.4%) patients achieved CR, 6 (3.2%) patients achieved CR without platelet recovery (CRi) or PR, and 82 (43.4%) patients did not achieve CR or had no evaluable CR data (Table 1). CR rates differed significantly between the two cohorts (85.1% in MDACC vs. 31.4% in NUH, *P* < 0.0001). Importantly, SAMHD1 expression levels did not correlate with response to induction therapy either in the entire study group of AML (*P* = 0.7583, Mann–Whitney *U* test) or in the subgroup analyses of only patients treated with HDAC during consolidation (*P* = 0.3203, Mann–Whitney *U* test). This is particularly important, as patients in the MDACC cohort all received HDAC during induction therapy. In addition, no differences for CR were found when analyzing patients for SAMHD1 expression that received ara-C-based treatment as an induction therapy either at NUH (*P* = 0.1241, Mann–Whitney *U* test) or at MDACC (*P* = 0.7659, Mann–Whitney *U* test), separately (Fig. 3).

### Prognostic significance of SAMHD1 expression levels in patients with AML treated with ara-C containing regimens

**Fig. 4:**
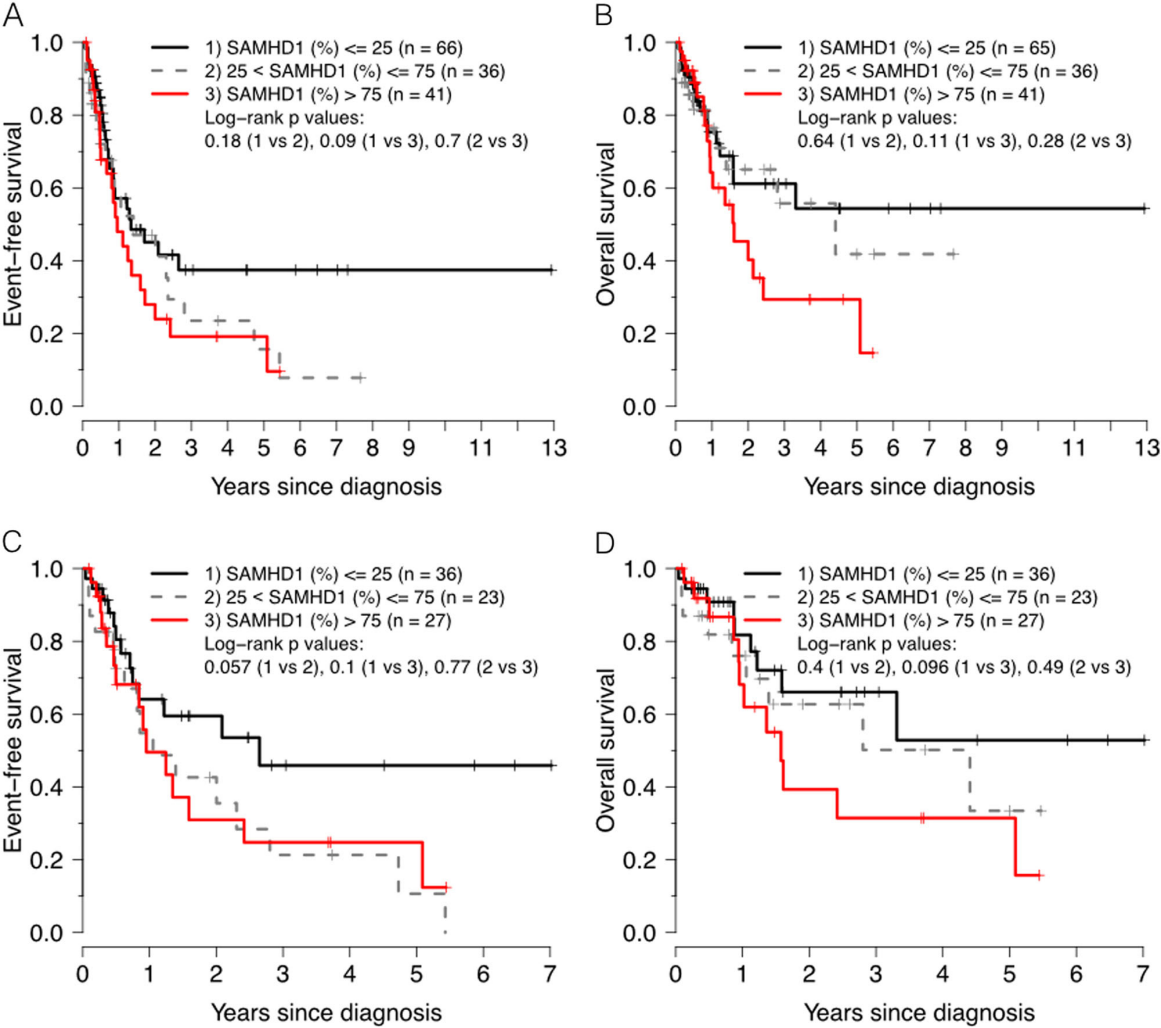
Kaplan–Meier curves showing EFS and OS of patients treated with ara-C-containing induction treatment: **a**, **b** all patients; **c**, **d** analysis restricted to intermediate risk group with diploid cytogenetics (MDACC cohort)

In the group of patients (*n* = 143) with available SAMHD1 and survival data receiving ara-C-based chemotherapy as an induction therapy, irrespective of their subsequent consolidation regimen, the 5-year EFS for patients with low versus high SAMHD1 levels was 37% versus 19%, respectively (*P* = 0.0896 by log-rank test) (Fig. 4a) after a median follow up of 36 months for survivors. Analyzing SAMHD1 expression as a continuous variable by multivariate Cox proportional hazards model revealed a hazard ratio of 1.008 for 1% increments (*P* = 0.022), and significance was maintained when adjusting for age, gender, risk group and FLT3-ITD status (*P* = 0.04), but not when adjusting for age, gender, risk group, and NPM1 status (Table 3). Similarly, the 5-year OS was 54 and 29%, for low versus high SAMHD1 levels, respectively (*P* = 0.107) (Fig. 4b) with a hazard ratio of 1.009 for 1% increments of SAMHD1 expression (*P* = 0.044), even though significance was lost in the adjusted analyses (*P* = 0.159 and *P* = 0.266) (Table 3).

**Table 3:** Multivariate analysis (Cox proportional hazards model) in the subsets of AML patients with Ara-C induction therapy and HDAC consolidation therapy: OS (HSCT-censored) and EFS (HSCT-censored)

		OS HSCT-censored			EFS HSCT-censored	
	AC ind.^a^	HDAC cons.^b^	AC ind. MDACC^c^	AC ind.	HDAC cons.	AC ind. MDACC
	HR^d^ (95% CI; *p*-value)	HR (95% CI; *p*-value)	HR (95% CI; *p*-value)	HR^d^ (95% CI; *p*-value)	HR (95% CI; *p*-value)	HR (95% CI; *p*-value)
Unadjusted	1.009 (1.000–1.017; 0.044)	1.014 (1.004–1.024; 0.007)	1.011 (1.000–1.022; 0.049)	1.008 (1.001–1.015; 0.022)	1.012 (1.004–1.020; 0.003)	1.009 (1.000–1.017; 0.049)
Adjusted for age, gender, risk group, FLT3-TKD	1.007 (0.997–1.018; 0.159)	1.010 (0.999–1.022; 0.078)	1.009 (0.998–1.020; 0.117)	1.008 (1.000–1.017; 0.040)	1.011 (1.002–1.020; 0.014)	1.009 (1.000–1.018; 0.039)
Adjusted for age, gender, risk group, NPM1	1.006 (0.995–1.017; 0.266)	1.008 (0.996–1.020; 0.196)	1.009 (0.997–1.021; 0.147)	1.008 (0.999–1.017; 0.075)	1.010 (1.000–1.020; 0.047)	1.008 (0.998–1.018; 0.102)

^a^Patients receiving AraC induction treatment

^b^Patients receiving high-dose AraC consolidation therapy

^c^Patients in the M.D. Anderson cohort receiving AraC induction therapy, not adjusted for risk group, due to all patients belonging to the intermediate risk group

^d^Hazard Ratio (HR) for a one-unit increase in SAMHD1 percent positive blasts

As SAMHD1 modulates efficacy of both clofarabine and fludarabine in THP-1 cells [8], we assessed whether SAMHD1 expression would have additional effects in patients receiving clofarabine or fludarabine in addition to HDAC-containing AML therapies by statistically evaluating interactions with SAMHD1. However, no significant interactions were detected (*P* = 0.755 and *P* = 0.811, respectively for overall survival) for the limited number of patients treated with clofarabine or fludarabine (*n* = 23, and *n* = 25, respectively) in the present study group.

**Fig. 5:**
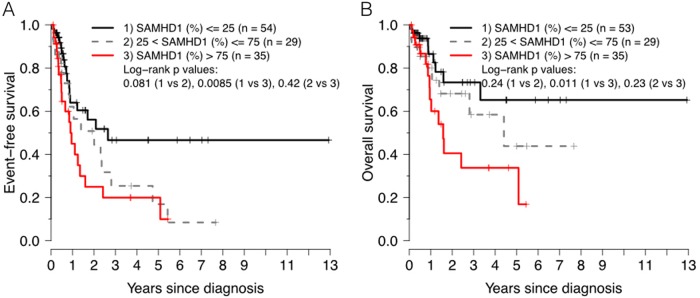
Kaplan–Meier curves showing event-free (**a**) and overall (**b**) survival of patients treated with HDAC consolidation therapy: all patients

When restricting the analysis to the group receiving HDAC as a consolidation therapy, the 5-year EFS for patients with low versus high SAMHD1 levels was 47% versus 20%, respectively (*P* = 0.00855 by log-rank test) (Fig. 5a) with a hazard ratio of 1.01 for 1% increments of SAMHD1 expression (*P* = 0.0032), and significance was maintained in adjusted analyses (*P* = 0.014 and *P* = 0.047) (Table 3). The corresponding 5-year OS was 44 and 34%, for low versus high SAMHD1 levels, respectively (*P* = 0.0114 by log-rank test) (Fig. 5b) with a hazard ratio of 1.01 for 1% increments of SAMHD1 expression (*P* = 0.0073) (Table 3), even though significance was lost in the adjusted analyses (*P* = 0.078 and *P* = 0.196).

Similar results were obtained when the analysis was restricted to the 87 patients in the intermediate risk group with diploid cytogenetics and available SAMHD1 data in the MDACC cohort (Table 3). Importantly, all of these patients were treated with HDAC consolidation regimens.

## Discussion

Here, we report for the first time the expression patterns of SAMHD1 in normal (reactive) bone marrows using an optimized double immunohistochemistry assay that analysed SAMHD1 expression in normal hematopoietic cells of all three lineages including normal CD34+ cells. Using this assay, no SAMHD1 expression was detected in normal MPO+ and GPA+ hematopoetic cells, megakaryocytes and normal CD34+ blasts. By contrast, SAMHD1 was strongly expressed in reactive, mature T-cells and histiocytes in reactive bone marrow specimens. Therefore, expression of SAMHD1 in myeloblasts of AML could be *bona fide* considered pathologic. Furthermore, this adds important information about expected excess ara-C myelotoxicity in possible clinical trials combining ara-C with putative SAMHD1 inhibitors.

In the present study, SAMHD1 expression as a continuous variable (% positive blasts) significantly correlated with WBC and LDH levels, but not with peripheral or bone marrow blast count and other clinicopathologic parameters. Future studies are needed to elucidate the biological role of SAMHD1 for turnover of AML blasts. Of note, SAMHD1 has anti-proliferative properties in vitro^13^.

Low expression levels of SAMHD1 protein as detected by immunohistochemistry were not associated with achievement of CR in two independent cohorts of AML patients of different risk groups in the present study, not even in the subgroup of patients that received HDAC as part of their induction therapy. Hence, our results suggest that therapy-limiting properties of SAMHD1 for HDAC therapies only become evident during post-remission consolidation. Notwithstanding, we are currently investigating whether SAMHD1 expression affects the rate of negative minimal residual disease (MRD) following ara-C-based induction courses as MRD significantly correlates with AML relapse. These findings are in accordance with the results of our recent study^7^ at the RNA level, where we showed, in both adult TCGA AML and the pediatric TARGET AML cohorts treated with ara-C, that *SAMHD1* mRNA levels are not lower in patients achieving CR as compared to patients that do not achieve CR. Hence, combined with the current study, no effect of SAMHD1 expression on CR is evident in four independent cohorts of AML patients.

By contrast, using immunohistochemistry, Schneider et al.^12^ reported a significantly lower expression of SAMHD1 in 112 AML patients who achieved CR as compared to a group of patients who did not achieve CR. Methodological confounders, like the use of a different SAMHD1 antibody (12586-1-AP from Proteintech, Rosemont, IL, USA) may account for this discrepancy^8,9,11^.

In the group of patients receiving ara-C-based chemotherapy as an induction therapy, irrespective of their subsequent consolidation regimen, Cox regression analyses indicated a significant effect of SAMHD1 expression on survival, however, the statistical power was lost when comparing high vs low expressers dichotomously. These data argue against a threshold effect defining a critical expression of SAMHD1 that confers ara-C resistance, consistent with our in vitro analyses using AML cell lines that showed a continuous, dose-dependent effect of SAMHD1 on ara-C sensitivity^7^. On the other hand, we showed that the protein levels of SAMHD1 are significantly associated with EFS and OS in patients treated with HDAC consolidation regimens, and these associations retained significance in the multivariate Cox proportional hazards model after adjustment with age, gender, risk group, and presence of FLT3-TKD or NPM1 mutations for EFS, but not OS, as to the loss of power is more pronounced for OS analyses with fewer events as compared to EFS. There were no significant correlations between SAMHD1 expression and these covariates. Larger future studies have to investigate whether SAMHD1 expression levels in specific subgroups of AML, such as the cohort of intermediate risk patients with diploid cytogenetics in the present study, may show stronger effects of SAMHD1 expression on survival.

In conclusion, we have shown that low-level SAMHD1 expression in AML blasts at diagnosis identifies a sizeable patient subset with favorable EFS and OS upon treatment with HDAC-containing consolidation regimens. As such, SAMHD1 expression, which can be assessed conveniently by immunohistochemistry, may represent a novel predictor of outcome in AML patients. Similarly, high SAMHD1 expression promises to be a predicitive biomarker for novel, selective SAMHD1 inhibitors which have been tested in our research laboratory with promising results^17^ that may lead to new, more efficient combination therapies.

## Electronic supplementary material

Supplementary figure 1

Supplementary figure 1 legend

Supplementary Methods
