# Supplementary figures and images for: Low-level expression of SAMHD1 in acute myeloid leukemia (AML) blasts correlates with improved outcome upon consolidation chemotherapy with high-dose cytarabine-based regimens
Source: Blood Cancer J. 2018 Oct 19;8(11):98. doi: 10.1038/s41408-018-0134-z (PMC6195559; doi:10.1038/s41408-018-0134-z)

A

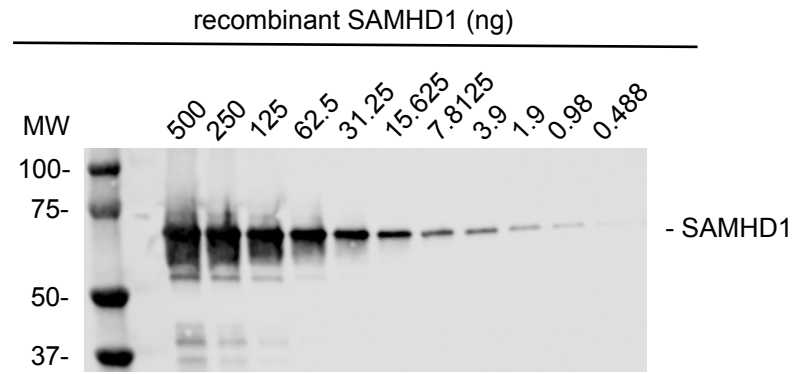

B

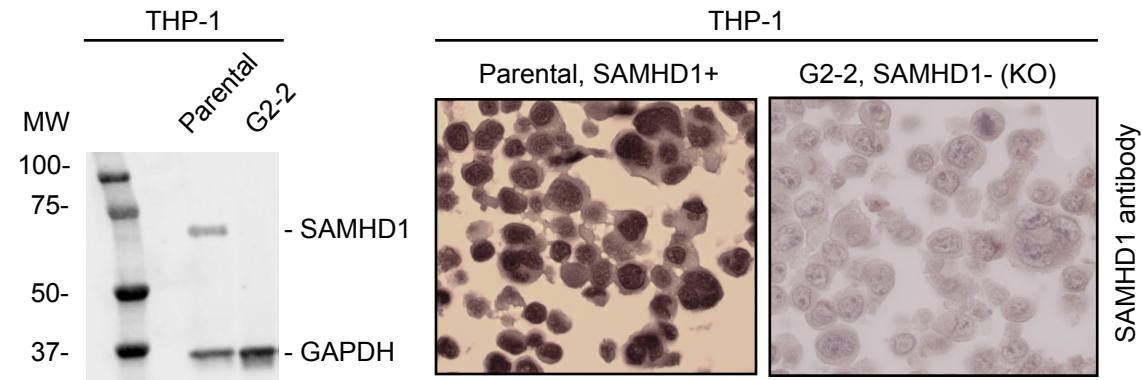

C

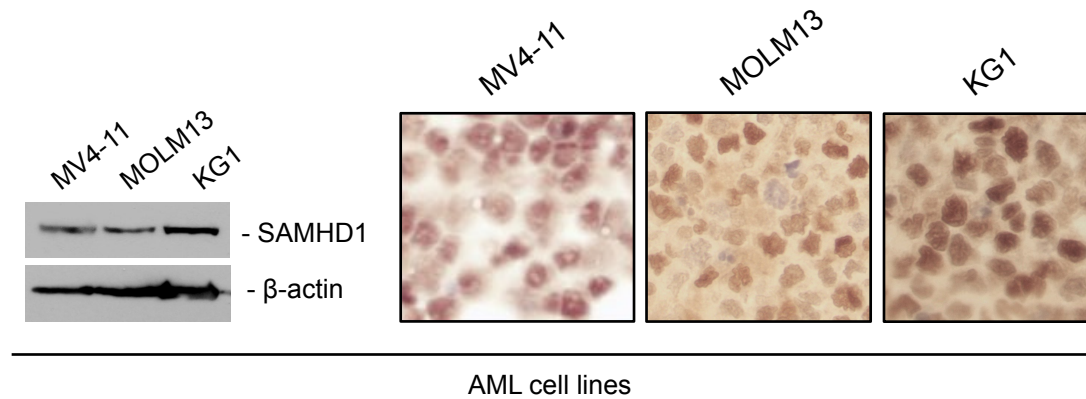

Supplement: Supplementary file 1 — Supplementary figure 1 [file 41408_2018_134_MOESM1_ESM.pdf]
