# Supplementary material for: Low-level expression of SAMHD1 in acute myeloid leukemia (AML) blasts correlates with improved outcome upon consolidation chemotherapy with high-dose cytarabine-based regimens
Source: Blood Cancer J. 2018 Oct 19;8(11):98. doi: 10.1038/s41408-018-0134-z (PMC6195559; doi:10.1038/s41408-018-0134-z)
Supplement: Supplementary file 2 — Supplementary figure 1 legend [file 41408_2018_134_MOESM2_ESM.docx]

**SUPPLEMENTARY FIGURE 1
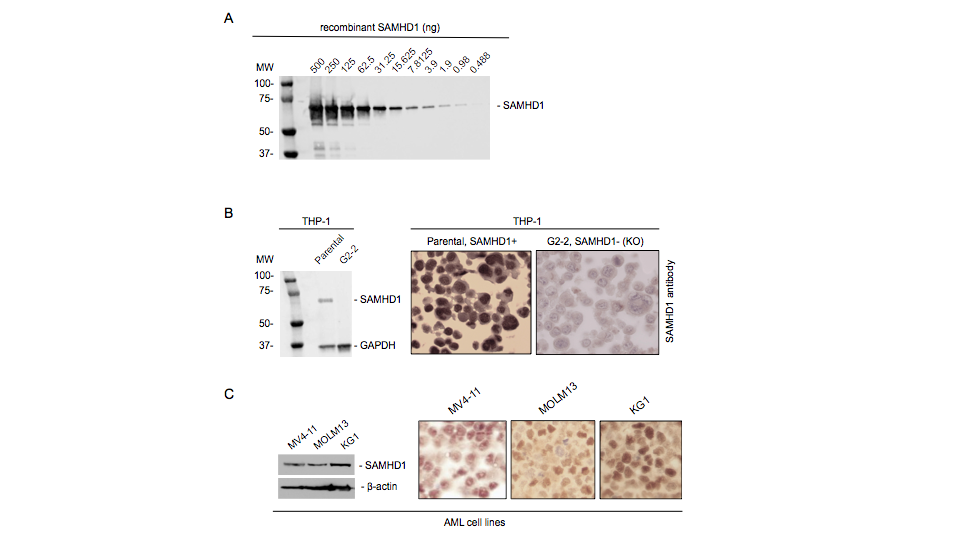
**

**Suppl. figure 1 legend**

The specificity and sensitivity of the SAMHD1 antibody were validated as follows:

**A**. Cels were transfected with decreasing concentrations of recombinant SAMHD1 and the SAMHD1 protein levels were detected by Western blot with the same antibody used in the immunohistochemical analysis. Decreasing levels of SAMHD1 protein were detected (from left to right) that parallel the levels of recombinant protein.

**B**. Parental THP-1 cells and their SAMHD1 knockout derivative G2-2 were generated by CRISPR/Cas9 as described previously (Herold et al, 2017, Nat Med). Whole lysates from both parental and G2-2 cells were prepared and Western blot analysis was performed as described in Materials and Methods. Primary antibodies used were anti-SAMHD1 (Bethyl laboratories, A303-691A) used at 1/2000, and anti-GAPDH (Santa Cruz, sc-47724) used at 1/2000). No SAMHD1 expression was detected in the knockout derivative G2-2 of THP-1 cells (left panel). Formalin-fixed, paraffin-embedded cell blocks were prepared from both parental and G2-2 THP-1 cells and histologic sections were subjected to immunohisto- chemical analysis using the same anti-SAMHD1 (Bethyl laboratories, A303-691A, used at 1/2000) and identical experimental conditions as for the immunostainings of the AML tissue microarray (TMA) sections. The parental THP-1 cells showed strong nuclear expression of SAMHD1, whereas no SAMHD1 expression is detected in the knockout derivative G2-2 of THP-1 cells (right panel).

**C.** In addition, expression of SAMHD1 was assessed in three AML cell lines including MV4-11, MOLM13 and KG-1 by Western blot analysis. A single band at the expected protein size was detected in all AML cell lines with KG-1 cells showing relatively higher SAMHD1 protein levels as compared with MV4-11 and MOLM13. Subsequently, formalin-fixed, paraffin-embedded cell blocks from the AML cell lines were prepared and the sections were immunostained with the same SAMHD1 antibody. Predominantly nuclear staining for SAMHD1 was detected in leukemia cells that parallel the levels of the SAMHD1 protein in immunoblots. For instance, KG-1 showed larger number of SAMHD1-positive cells and stronger staining intensity than MOLM13.
