# Supplementary material for: Low-level expression of SAMHD1 in acute myeloid leukemia (AML) blasts correlates with improved outcome upon consolidation chemotherapy with high-dose cytarabine-based regimens
Source: Blood Cancer J. 2018 Oct 19;8(11):98. doi: 10.1038/s41408-018-0134-z (PMC6195559; doi:10.1038/s41408-018-0134-z)
Supplement: Supplementary file 3 — Supplementary Methods [file 41408_2018_134_MOESM3_ESM.docx]

**SUPPLEMENTARY METHODS**

*Cell lines1*

Four AML cell lines including KG-1, MOLM13, MV4-11 and THP-1 were used. THP-1 cells and their SAMHD1 knockout derivatives generated by CRISPR/Cas9 have been described previously (1). All cell lines were grown in Roswell Park Memorial Institute (RPMI)-1640 medium (Life Technologies, Grand Island, NY, USA) supplemented with 10% fetal calf serum, and incubated at 37°C in a humidified atmosphere containing 5% CO2. Cell pellets were prepared using 20X10^6^ cells and the pellets were fixed in 4% formalin for 24 hours and then embedded in paraffin to make cell blocks. Sections were cut from the cell blocks and the sections were used for immunohistochemistry.

*Western blot analysis*

Cells were collected at exponential phase of growth and washed twice in cold PBS, and lysed at 4°C in lysis buffer as previously described (2). Western blot analysis was performed using standard method as reported elsewhere (2). The primary antibodies used were anti-SAMHD1 (cat. no. A303-691A, Bethyl Laboratories, San Antonio, TX, USA)) used at 1/2000, anti-GAPDH (cat. no. sc-47724 Santa Cruz, Dallas, TX, USA) used at a dilution of 1:2000, and β-actin (cat. no. A2228, Sigma, St. Louis, MO, USA ) used at a dilution 1:4000.

Purified recombinant SAMHD1 protein was prepared as described previously (1). Briefly, His-tagged SAMHD1 was expressed in *E. coli* BL21 Rosetta (DE3) pLysS (Novagen, Merck, Readington, NJ, USA) following addition of 0.5 mM IPTG and incubation at 17° C for 18 hours, and subsequently purified from lysate by chromatography on HisTrap and SP cation-exchange columns (GE Healthcare, Chicago, IL, USA).

REFERENCES

1. Herold N, Rudd SG, Ljungblad L, et al: Targeting SAMHD1 with the Vpx protein to improve cytarabine therapy for hematological malignancies. Nat Med 23:256-263, 2017.
2. Chen W, Drakos E, Grammatikakis I, et al: mTOR signaling is activated by FLT3 kinase and promotes survival of FLT3-mutated acute myeloid leukemia cells. Mol Cancer 9:292, 2010
